# Recent Advances in Biomolecular Detection Based on Aptamers and Nanoparticles

**DOI:** 10.3390/bios13040474

**Published:** 2023-04-13

**Authors:** Ruiting Xu, Leixin Ouyang, Heyi Chen, Ge Zhang, Jiang Zhe

**Affiliations:** 1 Department of Mechanical Engineering, University of Akron, Akron, OH 44325, USA; rx7@uakron.edu (R.X.); lo10@uakron.edu (L.O.); hc77@uakron.edu (H.C.); 2 Department of Biomedical Engineering, University of Akron, Akron, OH 44325, USA; ge10@uakron.edu

**Keywords:** aptamer, aptasensor, nanoparticles, fluorometry, colorimetry, electrochemical sensing, magnetic relaxation switching, resistive pulse sensor

## Abstract

The fast, accurate detection of biomolecules, ranging from nucleic acids and small molecules to proteins and cellular secretions, plays an essential role in various biomedical applications. These include disease diagnostics and prognostics, environmental monitoring, public health, and food safety. Aptamer recognition (DNA or RNA) has gained extensive attention for biomolecular detection due to its high selectivity, affinity, reproducibility, and robustness. Concurrently, biosensing with nanoparticles has been widely used for its high carrier capacity, stability and feasibility of incorporating optical and catalytic activity, and enhanced diffusivity. Biosensors based on aptamers and nanoparticles utilize the combination of their advantages and have become a promising technology for detecting of a wide variety of biomolecules with high sensitivity, reliability, specificity, and detection speed. Via various sensing mechanisms, target biomolecules have been quantified in terms of optical (e.g., colorimetric and fluorometric), magnetic, and electrical signals. In this review, we summarize the recent advances in and compare different aptamer–nanoparticle-based biosensors by nanoparticle types and detection mechanisms. We also share our views on the highlights and challenges of the different nanoparticle-aptamer-based biosensors.

## 1. Introduction

Biomolecules produced by living organisms and cells, including nucleic acids, proteins, carbohydrates, lipids, and metabolites [1,2,3], have a wide range of sizes and configurations and perform an array of biological functions. Monitoring and detecting these biomolecular analytes provide critical information for disease diagnosis, treatment efficacy, hematology, and pharmacology [4,5]. Increasing attention has been paid to the development of ultra-sensitive biosensors to achieve the fast, accurate, and real-time detection of biomolecules. Due to the different nature of biomolecules (e.g., dimensions, surface charges, mobility, etc.) [6,7,8,9], various detection methods have been developed. Commercially available instruments for biomolecular detection include surface-enhanced Raman spectroscopy (SERS) [10,11], surface plasmon resonance (SPR) [12,13], and gas chromatography-mass spectroscopy (GC-MS) [14,15]. SERS utilizes a special substrate to induce surface plasma resonances that enhance the Raman scattering signals from biomolecules. While SERS provides the sensitive detection of target biomolecules (typically at the nM level), it requires complex optical setup and instrumentation. Moreover, exposure to high-intensity laser beams can cause irreversible damage to bio-samples [16]. As an alternative method, SPR requires the immobilization of highly oriented antibodies on solid surfaces with the capability to resist non-specific protein adsorption [17,18]. Manipulating and controlling the orientation of antibodies and eliminating the non-specific adsorption of biomolecules from complex biological samples remains a challenge [18,19]. GC-MS is a commonly used method to identify and quantify volatile molecules based on a gas chromatograph analyzer coupled with a mass-selective detector. However, biomolecules are damaged during the measurement due to the separation and ionization of biomolecules. The bulky instrument and lengthy analysis process also make GC-MS unsuitable for on-site measurement. Currently, reverse transcription-polymerase chain reaction (RT-PCR) is widely used to detect nucleic acid segments, such as the genetic material of viruses [20]. RT-PCR provides highly sensitive and specific DNA (deoxyribonucleic acids) or RNA (ribonucleic acid) detection by amplifying segments of DNAs or RNAs [21], which are subsequently detected using molecular fluorophores. However, this method can only detect RNA or DNA segments with known sequences [22]. It has the risk of eliciting false-negative and false-positive results due to various reasons, such as improper sample collection and the presence of amplification inhibitors. Enzyme-linked immunosorbent assay (ELISA) is a well-established, gold standard method for detecting biomolecules, such as proteins, with high sensitivity and reliability. However, ELISA may not be applied to detect small biomolecules due to the limited surface area accommodated by antibodies [23]. Furthermore, ELISA requires labeling antibodies with fluorescent, enzymatic, or radioactive labels to form a sandwich structure around the target biomolecules. The measurement involves laborious and time-consuming procedures and skilled personnel [24,25]. Fluorescent detection typically needs to be performed in lab conditions with an optical reader. Further, the detection range is relatively narrow. Although the aforementioned methods have made impressive progress in the past decades, transformative improvements, such as ultra-high sensitivity, no tedious sample preparation, and a wider detection range, are highly desired.

Aptamers, single-stranded DNA, or RNA, can bind to specific biomolecules with high affinity [26,27]. Compared to antibodies, aptamers, a competitive alternative probe with high stability, have uniform activity regardless of the batch and an unlimited shelf-life and are not immunogenic. They can be produced through a simple and inexpensive process and are thus widely used in biosensors. One can also optimize the aptamer structure with the flexibility to achieve ultra-high sensitivity and selectivity for target analytes. Additionally, due to their small sizes, aptamers can achieve a high density of immobilization on the target surface [28]. Aptamer-based biosensors recognize target analytes via designed aptamers with high binding affinity and specificity and then convert biomolecular recognition into measurable physical or electrical signals via a readout component.

Nanoparticles (e.g., gold colloids, polymer nanoparticles, magnetic nanoparticles, etc.) are widely adopted for bioanalysis [29,30]. Nanoparticles have excellent biocompatibility, optical absorption and emission properties, or magnetic properties to amplify signals transduced from various biological mechanisms [20]. With a high surface-to-volume ratio, nanoparticles further contribute to the high immobilization capacity of aptamers. This contribution further enhances the performance of biomolecular recognition. Therefore, biosensors based on the combination of aptamer recognition and nanoparticles have been utilized for the rapid, specific, and accurate detection of various biomolecules, even at an ultra-low abundance.

**Figure 1:**
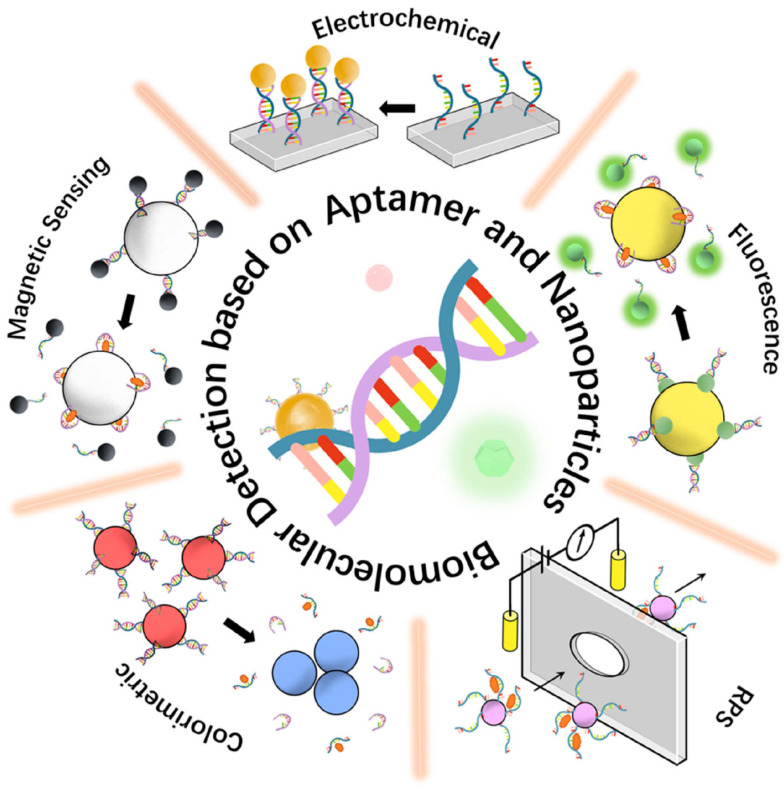
Schematic overview of different mechanisms of nanoparticle–aptamer-based biomolecular detection.

**Figure 2:**
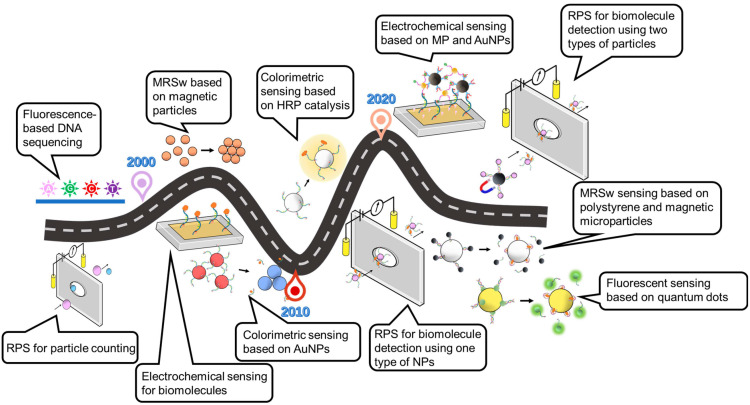
Roadmap/timeline of major milestones in nanoparticle–aptamer-based biomolecule sensors.

This review aims to summarize the latest advances in aptamer–nanoparticle-based biosensors. These aptasensors are reviewed by the types of nanoparticles (gold/magnetic/polystyrene nanoparticles) and detection mechanisms (colorimetric/fluorometric assay, magnetic relaxation switching, electrochemical assay, and resistive pulse sensing methods), and their advantages and limitations are outlined. The schematic to show the scope of this review work is shown in Figure 1. The timeline for nanoparticle–aptamer-based biomolecular sensors is shown in Figure 2.

## 2. Strategies for Nanoparticle-Based Aptasensors

### 2.1. Aptasensors Using Aptamer-Conjugated Gold Nanoparticles (AuNPs)

Gold nanoparticles (AuNPs) provide an ideal substrate for aptamer grafting owing to their excellent and unique properties (e.g., strong optical absorption and emission properties, chemical and electronic properties, high biocompatibility, inertness, etc.), which have attracted considerable attention in bioanalysis applications [31,32,33]. Colorimetric and fluorometric measurements in combination with AuNPs have been applied for biomolecular detection in various applications.

Colorimetric sensing based on metal AuNPs exhibits promising potential for clinical point of care due to its fast detection, low-cost, and simple instrumentation (e.g., naked-eye observation). It utilizes the intrinsic localized surface plasmon resonance (LSPR) effect to generate a colorimetric signal, i.e., the photons of light interacting with the AuNPs cause the free electrons to oscillate coherently at their resonant frequency. The LSPR results in localized electromagnetic field enhancement and wavelength-selective absorption with a strong extinction peak [34,35,36], which induces the spectral shift and color discrepancy of the AuNP solution. Due to the strong absorption and emission and biocompatibility (non-toxicity), AuNPs have become a powerful candidate for colorimetric sensing as signal transducers. Colorimetric sensing utilizes a suspension of aptamer-modified AuNPs of which the states are changed from being dispersed to aggregated in the presence of a target analyte. The state transition contributes to color transition owing to the coupling of LSPR that occurs when the distance between AuNPs is considerably less than the diameter of AuNPs due to the high degree of aggregation of AuNPs.

**Figure 3:**
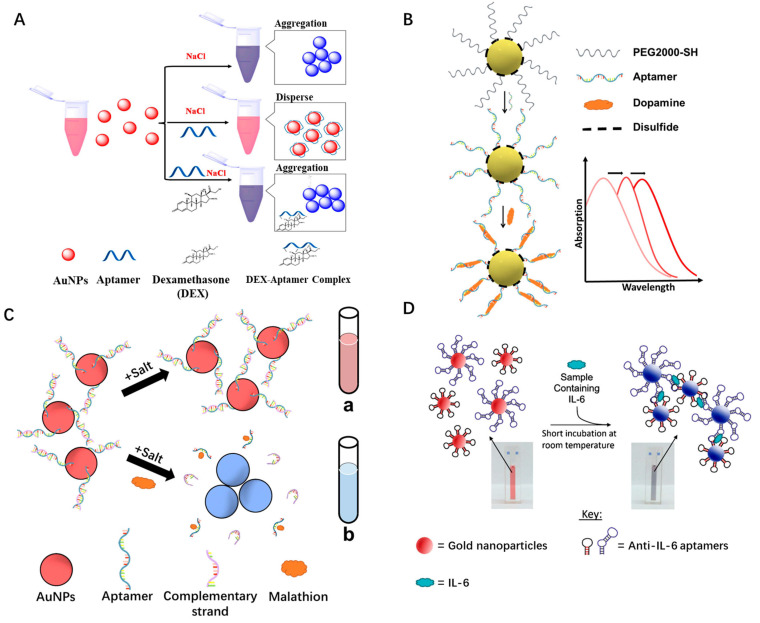
Colorimetric aptasensors based on AuNPs. (**A**) Schematic diagram of the colorimetric method for DEX detection, reproduced with permission from [37]. Copyright 2022 Biosensors. (**B**) Thiolated aptamer-modified AuNPs as recognition elements for the detection of dopamine. (**C**) Detection scheme of malathion based on dsDNA-modified AuNPs and hairpin structure of CS with (a) non-addition of targets and (b) addition of targets, causing the color change of solution. (**D**) Aptamer–AuNP-based aggregation assay for the detection of mouse IL-6, reproduced with permission from [45]. Copyright 2019 Microchimica Acta.

Figure 3A illustrates the mechanism of the colorimetric sensing method to detect target biomolecules [37]. The key mechanism for the transition of the AuNP state from dispersion to aggregation is based on the balance between interparticle electrostatic repulsion and attraction. The citrate-capped AuNPs prepared by the classic citrate reduction method are stabilized in water by charged citrate ions present on their surface. These charged ions create negatively charged electrical double layers on the surfaces of AuNPs, which generate electrostatic repulsive forces that prevent the AuNPs from sticking together via attractive van der Waals forces. However, the stability of the AuNPs is strongly influenced by the strength of the electric double layer, which depends on the salt concentration in the solution. When the salt concentration is high, the counter ions in the solution reduce the electric charge on the citrate ions, leading to a weak electric double layer. As a result, the repulsive force between the AuNPs diminishes; the attractive van der Waals forces become more dominant, which leads to the aggregation of AuNPs. When aptamers are grafted on the surface of AuNPs, they create a barrier that prevents (1) AuNPs from coming too close and (2) van der Waals attractive forces from dominating. The stability of aptamer-modified AuNPs is less influenced by the ionic strength and more by the molecular weight of the aptamer and surface graft density [38,39]. Once target analytes were added, the analytes had a higher affinity with aptamers than AuNPs, resulting in the decomposition of aptamer–AuNP complexes. The bare AuNPs aggregated again from their lack of stability in the suspension, changing the color of the suspension from red to blue. By optimizing the sensing conditions (e.g., AuNP size, salt type, and concentration), one can achieve the rapid detection of targets by monitoring the color variation [37]. Lerga et al. [40] optimized the sensing conditions by using different types and concentrations of salt (e.g., NaCl, KCl, or MgCl_2_) and AuNP sizes (ranging from 10 nm to 60 nm) for the detection of histamine. After optimizing sensing conditions, the limit of detection (LOD) using 16 nm AuNPs was 8 nM at a 60 mM NaCl concentration, and the detection range of histamine was 0–2000 nM. One critical limitation of this sensing method is the incomplete dissociation of non-target binding fragments of aptamers on the surface of AuNPs, which causes the inability of AuNPs to aggregate even with the addition of salt solution, eventually resulting in false-positive results. Alsager et al. [41] introduced a centrifugation and resuspension step to eliminate the residual binding between aptamer and AuNPs. This procedure was validated by detecting vitamin D3 under the optimized sensing conditions. The performance of the aptamer-based sensing was enhanced with a detection limit of 1 nM. Furthermore, the immobilization method of aptamers on the surface of AuNPs is also critical to prevent the non-specific aggregation of AuNPs. With the conventional method, AuNPs were modified by aptamers via physical absorption. Yano-Ozawa et al. [42] utilized thiolated DNA (DNA_brush_, Au-S bonds) to modify AuNPs at a high density, resulting in the significant suppression of non-target aggregation. To compare DNA_brush_-AuNPs and DNA_adsorbed_-AuNPs (physical adsorption), a non-target (antibiotic kanamycin) was tested. It induced the aggregation of DNA_adsorbed_-AuNPs, but no aggregation of DNA_brush_-AuNPs was found. Moreover, the effect of different lengths of DNAs (thio18-T5 with 13 bases, thio18 with 18 bases, and thio18+T5 with 23 bases) was evaluated with the same amount of thiol DNAs. AuNPs modified by longer thiolated DNA bases exhibited more stability, indicating that more negative charges contributed to the prevention of non-target aggregation. Finally, a specific complementary aptamer was hybridized with DNA_brush_-AuNPs for the determination of estradiol (E2) as a proof of concept. After the addition of E2, the detachment of aptamers from the DNA_brush_ resulted in the formation of the aptamer–E2 complex, causing the aggregation of AuNPs and a color change. However, if the next batch of samples needs to be tested, AuNPs need to be remodified by aptamers. The sensing platform is unable to be reused without the time-consuming surface remodification.

To address this issue, Niyonambaza et al. [43] proposed a reusable sensor for dopamine detection, as shown in Figure 3B. The AuNPs were modified with desalted thiolated aptamers via strong thiol-gold linking (Au-S). After modification, the plasmon band center shifted from 513 nm to 549 nm due to the change in the hydrodynamic diameter of AuNPs from 11.7 ± 0.3 nm to 13.5 ± 0.3 nm. In the presence of dopamine, the hydrodynamic diameter of the dopamine-AuNP complex was about 15.9 ± 0.3 nm and exhibited an increased absorbance of a longer wavelength, resulting in a bathochromic shift to 570 nm. The detection assays demonstrated a noticeable linear relationship between dopamine concentrations and the plasmon shift. Unlike the target-induced aggregation in traditional aptasensors, the targets can be filtered from the detection system due to the dynamic binding between the aptamer and target using centrifugation (Amicon^®^Ultra 0.5 mL centrifugal filters) at 14,000× *g* about 7–8 times. The recycled AuNP solution exhibits a similar response to targets when compared to that of freshly synthesized AuNPs. This method provided a ultra-stable and reusable platform for the highly selective detection of dopamine. Furthermore, AuNPs can also be modified by aptamer/complementary strand conjugation as a double-stranded DNA (dsDNA) structure via Van der Waals forces and electrostatic interaction. This structure can mitigate non-specific, salt-induced aggregation, and improve assay sensitivity and stability (see Figure 3C-a). When the targets were added, aptamers recognized and bound target molecules, releasing the complementary strand (hairpin structure) and aptamer–target complexes. Due to the rigid nature of the hairpin structure, the released complementary strands were not captured by AuNPs, causing the salt-induced aggregation of AuNPs (See Figure 3C-b). The dsDNA aptamer structure not only protected AuNPs from salt-induced aggregation, but also achieved a lower detection range and higher sensitivity, as shown in Figure 3C. Abnous et al. [44] utilized this mechanism to determine pesticide malathion with a lower detection range of 5 pM to 10 nM and a higher sensitivity of 1 pM. It is worth mentioning that the prior detection methods all required a long incubation time. Giorgi-Coll et al. [45] used a sandwiched structure for the determination of interleukin-6. Two anti-IL-6 aptamers were used to modify AuNPs. Interleukin-6 (IL-6) targets were recognized by the two aptamers and bound to them at different sites, forming sandwiched aggregates of AuNPs, which exhibited a color transition (see Figure 3D). The formation of the sandwiched aggregates was more rapid compared to that with the previously mentioned strategies. The simple operation of this method enables on-site detection with the induced color change visible within 5 min. For interleukin-6, this sensor had a detection range from 3.3 to 125 μg/mL and an LOD of 1.95 μg/mL. Currently, this colorimetric sensing method is applied not only for detecting small biomolecules but also for bacteria detection (e.g., *Pseudomona* aeruginosa) with a decent LOD [46].

**Figure 4:**
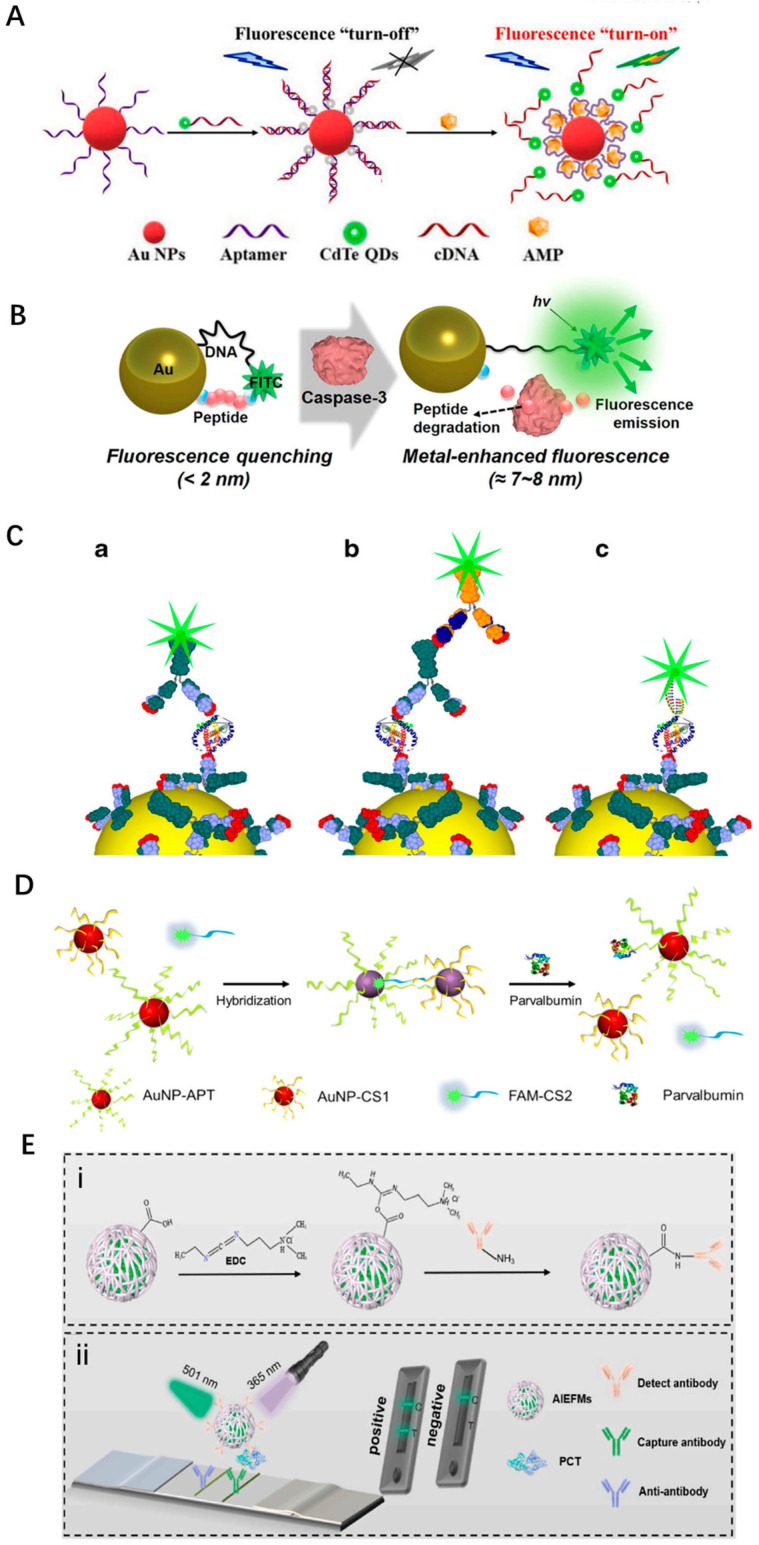
Fluorometric Apta-biosensing methods based on AuNPs. (**A**) A turn-on fluorescent aptasensor for AMP detection, reproduced with permission from [49]. Copyright 2022 Microchemical Journal. (**B**) MEF-based aptasensor for caspase-3 detection, reproduced with permission from [55]. Copyright 2020 American Chemical Society. (**C**) Schemes of a MET-based aptasensor for pfLDH detection: (a) Antibody–Target–Antibody, (b) Antibody–Target–Antibody–Antibody, and (c) Antibody–Target–Aptamer, reproduced with permission from [56]. Copyright 2021 Microchimica Acta. (**D**) Dual-mode aptasensor for PV detection based on state transition of AuNPs and fluorescence variation of FAM-CS2, reproduced with permission from [57]. Copyright 2020 Microchemical Journal. (**E**) Schematic of PCT detection with a sandwich format. (i) The synthesis of AIEFM@mAbs. (ii) The illustration of sandwich format by using green emissive AIEFMs, reproduced with permission from [59]. Copyright 2023 Analytica Chimica Acta.

While colorimetric aptasensors have the advantages of cost-effectiveness, a fast response, and ease of observation by the naked eye, the sensitivity and LOD need further improvements. Fluorescence detection is one of the primary analytical methods in biosensing. A fluorescent tag, such as a quantum dot, can serve as a reporter ‘flare’. In this mechanism (Figure 4A), high-density single-stranded (ss) aptamer-functionalized AuNPs were used to circumvent salt-induced aggregation and provide excellent stability. Fluorophores, Graphene Quantum Dot (gQD), conjugated by the complementary sequence (CS) of the aptamers were quenched by aptamer-modified AuNPs, forming an AuNP–aptamer–gQD complex. Target analytes, which have a higher affinity for the aptamers than the complementary sequences, were bound to the aptamers, resulting in a conformational change that disrupts the pairing between the aptamer and the CS-conjugated fluorophore. Eventually, the fluorophores were released from the surfaces of AuNPs [47,48]. The emitted fluorescent intensity from fluorophores was proportional to the concentrations of target analytes. Note that photobleaching typically occurs under a brief high-intensity light exposure that stimulates fluorophores to fluoresce. The exposure prevents fluorophores from permanently fluorescing within the illuminated area and results in decreased fluorescent intensity in the illuminated region. The fluorescent intensity can be recovered by allowing for the sufficient diffusion of unbleached fluorophores into the illuminated region. Several researchers utilized aptamer–AuNP-based fluorometric sensing mechanisms to realize the detection of biomolecules with high detection limits or high fluorescence recovery rates. Yu et al. [49] designed a fluorescent aptasensor for ampicillin (AMP) detection by utilizing CdTe quantum dots (QDs) and AuNPs (see Figure 4A). Aptamer-modified AuNPs hybridized with CS-functionalized CdTe QDs to form the complexes. In the presence of AMP, the CS-conjugated QDs dissociated from the complexes due to the strong binding between AMP and the aptamer, resulting in increased fluorescence intensity. By optimizing the concentration of AuNPs, a good fluorescence-recovery effect was demonstrated. The aptasensor achieved a good linear detection range of 0.04–20 μM and an LOD of 18 nM with a recovery rate of 88.4–99.0% for AMP detection. Wang et al. [50] proposed a fluorometric method for aflatoxin B1 (AFB1) detection by combining CS-conjugated AuNPs and a fluorescein-labeled aptamer. Aptamers hybridized with CS on the surface of AuNPs, causing fluorescence quenching. In the presence of AFB1, AFB1 prefers to bind with aptamers instead of CSs, causing the recovery of fluorescence. This aptasensor achieved an AFB1 detection range of 61 pM to 4.0 μM with an LOD of 61 pM. Furthermore, to achieve higher sensitivity, key variables, such as the length of the aptamer and the hybridization site and length, were also designed and optimized. Sun et al. [51] optimized these parameters by detecting rHuEPO-α and achieved nanomole-level sensitivity (0.92 nM). Although fluorescence-based sensors have been well established, the long incubation and detection times (usually a few hours) cannot be ignored, especially in the presence of background interference from complex biological systems [52]. Sensitivity also can be further improved by enhancing incident fluorescent intensity.

Metal-enhanced fluorescence (MEF) has been used to amplify the fluorescent intensity when the fluorophores are near a noble-metal nanostructure, which dramatically enhances the emission and excitation intensity due to the resonant coupling between them [53,54]. JH Choi and JW Choi [55] proposed a fast, simple technique for the real-time detection of intracellular proteolytic enzymes (caspase-3), as shown in Figure 4B. AuNP and fluorescein isothiocyanate (FITC) were connected by a double bridge (a long aptamer sequence and a short specific peptide sequence). The short peptide could be cleaved selectively by caspase-3. Subsequently, AuNP and FITC were connected by a single aptamer sequence after the peptide cleavage reaction. The distance between the AuNP and FITC was increased. An enhanced fluorescent signal was emitted from the FITC-aptamer–AuNP due to the MEF effect. They also optimized the aptamer length based on the number of thymine bases ranging from 0 to 30. The optimal aptamer length was ~7 nm with 20 nucleotides for inducing an efficient MEF effect. The sensor achieved the rapid detection (<1 h) of caspase-3 and a detection range from 10 to 10,000 pg/mL. Since AuNPs were dispersed freely in the solution, the fluorescent intensity was affected by the distance between the position of the fluorophore and the detector. Minopoli et al. [56] utilized the photochemical immobilization technique (PIT) to immobilize AuNPs onto a glass substrate through electrostatic self-assembly, as shown in Figure 4C. Three feasible configurations were compared to determine the optimal distance between the AuNP and fluorophore, including Antibody–Target–Antibody, Antibody–Target–Antibody–Antibody, and Antibody–Target–Aptamer. The scheme of the Antibody–Target–Aptamer was used to carry out the detection of PfLDH (Plasmodium falciparum lactate dehydrogenase) with optimal distance for the MEF phenomenon (approximately 10–15 nm). In this structure, the target biomolecule (PfLDH) was bound with both antibody-modified AuNPs and fluorescently labeled aptamers, forming a sandwiched structure. As MET with the optimized distance enhanced fluorescent intensity, the proposed biosensor achieved rapid detection (30 min incubation time) without sample pretreatment and an LOD of 10 pM (0.3 ng/mL). Considering the fast detection of colorimetric sensing and high sensitivity detection of fluorometric sensing, a dual-mode aptasensor based on both colorimetric and fluorescent readouts was developed, with the potential advantages of high sensitivity, naked-eye monitoring, and easy and fast operation. Wang et al. [57] proposed a dual-mode aptasensor for parvalbumin (PV) detection (see Figure 4D). In this strategy, aptamer-modified AuNPs (AuNP-APT) were hybridized with short complementary sequence 1 (CS1)-conjugated AuNPs (AuNP-CS1) and complementary sequence 2 (CS2)-conjugated fluorescent dye (FAM-CS2). CS1 and CS2 bound with APT (aptamer) at different sites via hybridization. The presence of PV triggered the dissociation of complexes via the competitive interaction between PV and the aptamer, resulting in the state transition from aggregation to the dispersal of AuNPs and increased fluorescent intensity. The state transition also caused the absorption of different wavelengths and a color discrepancy. The increased fluorescent intensity was measured using a microplate reader, and the color change could be visually identified. This aptasensor demonstrated quantitative analysis of PV with a colorimetric detection range of 2.5–20 μg/mL and a fluorometric detection range of 2.38–40 μg/mL. However, for traditional fluorescent dyes, due to the aggregation-caused quenching (ACQ) effect, especially at high concentrations, the sensitivity and stability of fluorescent dyes can be affected seriously, causing a low signal-to-noise ratio and photobleaching [58]. To overcome these problems, Wei et al. [59] utilized aggregation-induced emission luminogen (AIEgen)-embedded fluorescent microspheres (AIEFMs) instead of fluorescein dyes for the detection of procalcitonin (PCT), as shown in Figure 4E. For the synthesis of AIEFM@mAbs, the AIEFM reacted with EDC (1-ethyl-3-(3-dimethylaminopropyl) carbodiimide) firstly, and then the carboxyl groups of AIEFMS coupled with the amino groups of the detected antibody (anti-PCT mAbs) to serve as the signal reporter (see Figure 4E i). The nitrocellulose (NC) membrane was modified by two kinds of antibodies, anti-PCT pAbs (capture antibody at T line) and goat anti-mouse IgG (anti-antibody at C line). When the PCT sample solution was added, the capture antibody and detection antibody both bound to PCT and formed a capture antibody–PCT–detected antibody “sandwich” structure, causing a green fluorescent band on the T line. Then, the sample solution passed through the T line; the detected antibodies were captured by anti-antibodies on the C line to emit a fluorescent band (see Figure 4E(ii)). The fluorescent intensity was recorded by a commercial strip reader. The AIEFMs exhibited a stronger fluorescent intensity and achieved a dynamic linear detection of PCT from 7.6 pg/mL to 125 ng/mL with an LOD of 3.8 pg/mL.

### 2.2. Biosensing Based on Magnetic Particle (MP) and Gold Nanoparticle (AuNP) Complexes

Magnetic particles (MPs) also show excellent potential for biosensors with high chemical stability, a highly active surface, and ease of surface modification [60,61]. Their magnetism contributes to the separation and immobilization of targets under a magnetic field. Additionally, MPs can amplify aptamer-based target recognition due to loaded high-density signal tags and accelerate signal transduction among catalytic activities, thus improving sensitivity and selectivity [62]. These merits make MPs widely used for cost-effective, reliable biosensors [61].

#### 2.2.1. Aptamer Capture Assays for Biomolecules Based on MP and AuNPs

**Figure 5:**
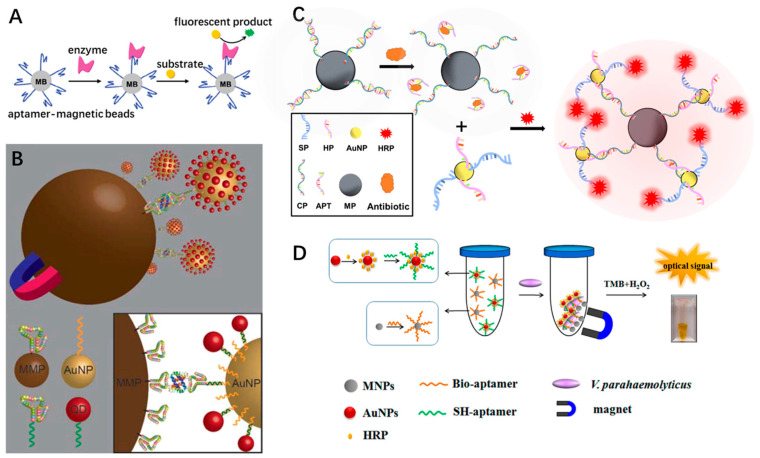
Aptamer-capture assay methods via fluorometric and colorimetric sensing. (**A**) Schematic diagram of the aptamer–enzyme biosensor for enzyme detection, reproduced with permission from [63]. Copyright 2011 American Chemistry Society. (**B**) Aptamer-based sandwich assay for malaria antigen detection, reproduced with permission from [65]. Copyright 2017 American Chemistry Society. (**C**) A colorimetric aptasensor based on signal amplification for the detection of antibiotics. (**D**) A colorimetric aptasensor based on HRP for V. parahaemolyticus detection, reproduced with permission from [68]. Copyright 2015 American Chemical Society.

As shown in Figure 5A, Zhao et al. [63] developed a simple sensing method that utilized aptamer-modified MPs as affinity probes to capture and isolate target enzymes. These captured enzymes catalyzed the transition of the fluorogenic substrate to fluorescent products. Two enzymes (2 fM human α thrombin and 100 fM human neutrophil elastase, HNE) were tested to demonstrate the feasibility of the sensor. To shorten the enzyme reaction time (3 h at 37 °C), Tennico et al. [64] utilized quantum dots to replace the fluorogenic substrate for thrombin detection. Two types of thrombin aptamers were selected to recognize two different epitopes of thrombin. MPs modified by one type of aptamer were immobilized on the magnetic substrate. Quantum dots were functionalized by the other type of aptamer serving as the signal probe. Due to the presence of thrombin, two types of aptamers bound to the target together. Quantum dots were detected via fluorescence microscopy. The total incubation and reaction time was reduced to ten minutes, and the fluorescent intensity was measured to determine the presence and concentration of the target. This sensor achieved an LOD of 10 ng/mL with an average standard deviation of 8% in accuracy for thrombin detection. For this method, only one quantum dot fluorophore was conjugated to one target molecule via one aptamer (one-to-one binding mode), resulting in relatively low detection sensitivity. The fluorescent signal amplification is necessary for increasing the sensitivity. Kim and Searson [65] proposed an ultrasensitive method by conjugating multiple quantum dots onto the surface of AuNPs for signal amplification via only one aptamer, as shown in Figure 5B. The sensor consists of aptamer-modified magnetic microparticles (MMPs) and AuNP–quantum dots. Due to the presence of the target antigen, aptamer-modified MMPs bound with AuNP–quantum dots. By utilizing AuNPs conjugated with multiple quantum dots, for Plasmodium lactate dehydrogenase (pLDH), this method achieved an LOD of 10 aM in 100 μL (corresponding to 1 pg/mL). Additionally, the aptamer-based biosensors that combined quantum dot-conjugated AuNPs and MMPs can also detect the specific surface-bound proteins on the membranes of bacteria (e.g., anti-Salmonella typhimurium antibodies). Thus, these biosensors can detect bacteria with high specificity and sensitivity (e.g., detection range of 10–10^7^/mL, LOD of 13.6/mL for Salmonella typhimurium, S. typhi) [66]. Although fluorometric sensing provides highly sensitive and specific detection of the target, it requires bulky optical instrumentation. Colorimetric sensing with low-cost, stable, and visual sensing is a potential alternative. However, the sensitivity of traditional colorimetric sensing is relatively low. Xu et al. [67] integrated aptamer-modified magnetic particles (MPs) and AuNPs for the determination of oxytetracycline (OTC) and kanamycin (KAN), as shown in Figure 5C. In this strategy, AuNPs were co-functionalized by the signal probe (SP) and the help probe (HP) via Au-S chemistry, where SPs were tagged by horseradish peroxidase (HRP). The biotin-modified capture probe (CP) was hybridized with an aptamer probe (APT) to form the dsDNA duplex that was attached to the surface of MPs. The presence of target antibiotics (OTC or KAN) triggered the disassociation of the aptamer sequence (APT) from the surfaces of MPs, resulting in the release of the aptamer–antibiotic complex. The remaining CP hybridized with the HP of AuNPs so that HRP was captured by the MP–Au system. The colorimetric response was generated after the catalytical oxidization reaction of 3,3′,5,5′-tetramethylbenzidine (TMB) and o-phenylenediamine (OPD) with HRP. This work demonstrated an extremely wide detection range (from 10^−6^ to 10^5^ pg/mL) and a very high LOD of 1 ag/mL for KAN and OTC. Wu et al. [68] utilized the AuNP–HRP–aptamer-target–aptamer–MNP system for the detection of Vibrio parahaemolyticus (V. parahaemolyticus), as shown in Figure 5D. Bio-aptamer-modified MNPs served as capture probes, while a large amount of HRP and SH-aptamer-modified AuNPs served as signal amplifiers. In the presence of V. parahaemolyticus, two aptamers bound to different transmembrane proteins to form the complexes. Subsequently, HRP catalyzed TMB-H_2_O_2_, resulting in color variation. Under optimal conditions, this method had a detection range of 10 to 10^6^ colony-forming units (cfu)/mL and an LOD of 10 cfu/mL. In addition, aptasensor-based colorimetric sensing also achieved cell detection with high sensitivity (detection range of 10–10,000 cells/mL, LOD of 3 cells/mL for circulating tumor cells, MCF-7) [69].

**Figure 6:**
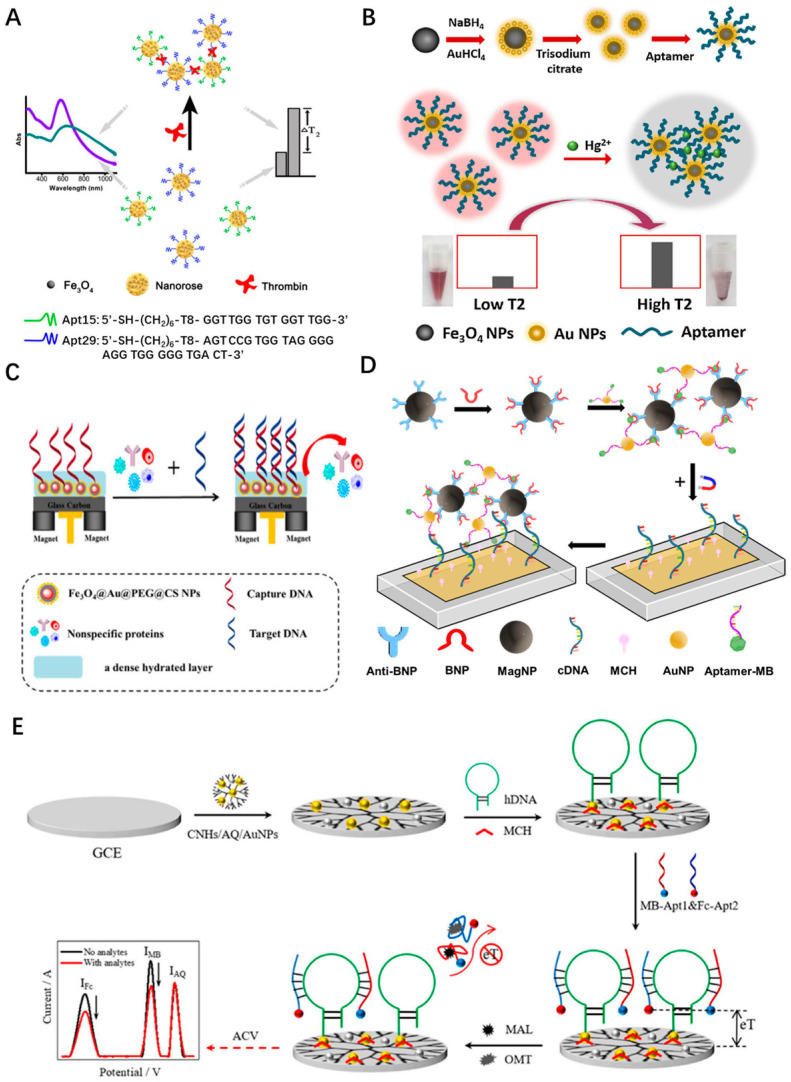
Aptamer capture assay methods. (**A**) Aptamer-linked sandwich assay for thrombin detection using Fe_3_O_4_@Au nanoparticles, reproduced with permission from [71]. Copyright 2011 Analytica Chimica Acta. (**B**) The dual-mode sensor for Hg^2+^ detection based on MRSw and colorimetric sensing, reproduced with permission from [72]. Copyright 2020 Journal of Hazardous Materials. (**C**) The low fouling electrochemical biosensor for Mycoplasma ovipneumoniae detection, reproduced with permission from [75]. Copyright 2020 Analytica Chimica Acta. (**D**) The triple recognition electrochemical immunoassay based on functionalized nanoparticles for BNP detection. (**E**) Schematic of dual-ratiometeric electrochemical aptasensor based on CNHs/AQ/AuNPs composites, reproduced with permission from [78]. Copyright 2023 Talanta.

Optical aptasensors show great potential in environmental and biological fields with high sensitivity and reliability. However, the detected signals can be affected by signal bleaching, background interference, and light scattering from the samples [70]. A magnetic relaxation switching (MRSw) assay provides a promising method for addressing these issues. The mechanism utilizes the target-induced aggregation (or dispersion) of magnetic nanoparticles (MNPs). Subsequently, the spin-spin relaxation time (T_2_) of water protons is changed and detected via nuclear magnetic resonance. In addition, this method does not need to isolate aggregations from the free MNPs; the signal can be obtained from the entire volume of the sample. Liang et al. [71] proposed a sensitive biosensing system integrating MRSw and colorimetry for detecting human α-thrombin, as shown in Figure 6A. In the presence of thrombin, two aptamer-modified Fe_3_O_4_@Au NPs (gold-coated iron oxide nanoparticles) aggregated due to target recognition, resulting in a change of T_2_; color transition occurred subsequently due to the aggregation of Fe_3_O_4_@Au. A shift in the UV-Vis absorption spectra was also detected. The dual-mode sensor achieved a detection range of 1.6 nM to 30.4 nM and an LOD of 1.0 nM. Liu et al. [72] demonstrated a dual-mode sensor that achieved a wider detection range by utilizing AuNP-coated Fe_3_O_4_ combining MRSw, as shown in Figure 6B. The presence of Hg^2+^ triggered the aggregation of aptamer-modified Au@Fe_3_O_4_ (Fe_3_O_4_ coated with AuNPs) and thus a T_2_ shift. This sensor had a wider detection range of 10 nM to 5 μM with an LOD of 2.7 nM. Moreover, an aptamer-functionalized MRSw sensor can be used for detecting pathogens (e.g., Vibrio alginolyticus) in terms of recognizing the specific ligand on the membrane [73]. Aptamer-modified MNPs (Fe_3_O_4_@SiO_2_-NH_2_) interacted with Vibrio alginolyticus, leading to a different T_2_. Wang et al. [73] also optimized the conditions (i.e., the concentrations of MNPs and aptamers) and obtained an LOD of 26 cfu/mL and a detection range of 4 to 4 × 10^3^ cfu/mL for the detection of Vibrio alginolyticus. For aptamer-functionalized MRSw sensors, several factors, including the sizes and concentrations of MNPs, the number of binding sites on the MNPs, and recognition elements (e.g., selection of aptamers), affect the sensitivity and the detection range. Thus, the optimization of these factors is a prerequisite for detection.

While the aforementioned magnetic relaxation methods demonstrate high sensitivity for biomolecule detection, these methods rely on sophisticated instrumentations and trained technicians, which limit the potential applications for point-of-care (POC). An electrochemical immunoassay exhibits great potential for POC testing with sensitive, portable, cost-efficient, and fast analysis [74]. Zhao et al. [75] developed an electrochemical biosensor for mycoplasma ovipneumoniae (MO) determination, as shown in Figure 6C. Gold-coated magnetic particles (Fe_3_O_4_@Au) used in this biosensor have excellent electrical conductivity, chemical-modification ability, and stability. Fe_3_O_4_@Au NPs were immobilized on the magnetic glassy carbon electrode by a magnetic field. When specific aptamers representing MO were added, they were anchored on the surface of Fe_3_O_4_@Au. Electrochemical measurements (cyclic voltammetry, differential pulse voltammetry, and electrochemical impedance spectroscopy) were used to record the change of the current as the electrochemical signals. Various molecules (WB-DNA, M1-DNA, M2-DNA, M3-DNA, and Target DNA) were tested with corresponding capture aptamers immobilized on the surfaces of Fe_3_O_4_@Au NPs to demonstrate the selectivity of the biosensor. The testing demonstrated that the biosensor was highly selective, which only exhibited a strong current change in response to the target DNAs. Next, target DNAs with low concentrations (from 10^−18^ M to 10^−12^ M) were added to the whole serum to represent a complex environment. The biosensor still had high analytical performance with an LOD of 3.3 aM. To further improve the sensitivity of the electrochemical sensor, the detected electrochemical signal needs to be amplified. Zhao et al. [76] proposed a triple recognition voltametric method for the detection of brain natriuretic peptides (BNP) (see Figure 6D). Methylene blue-labeled aptamer (Apt-MB)-modified AuNPs and anti-BNP-modified magnetic nanoparticles (MNPs) were utilized as signal amplifiers and reporters. In the presence of BNP, anti-BNP-functionalized MNPs and AuNPs were conjugated via specific interactions between the C-terminal of BNP and the aptamer. Then, the MNP–AuNP nanocomposites were captured by the complementary sequence’s modified gold electrode surface via hybridization between Apt-MB and complementary sequences, thereby amplifying the electrochemical signal (current change) due to the captured AuNP–MNP complexes. Zhao et al. also optimized experimental parameters, such as the antibody concentration (20 μg/mL), pH (7), temperature (37 °C), and reaction time (20 min). Under the optimal conditions, the results demonstrated a linear detection range of 1–10,000 pg/mL with an LOD of 0.56 pg/mL and a <6% standard deviation in accuracy. Wang et al. [77] proposed an electrochemical aptasensor for the rapid, on-site quantification of bacteria, such as *vibrio parahaemolyticus* (V.P). An aptamer-modified magnetic nanoscale metal-organic framework (Fe_3_O_4_@NMOF), used as capture probes, was attached to a screen-printed electrode (SPE) via an external magnet. Phenylboronic acid (PBA) and ferrocene (Fc) were co-functionalized on AuNPs (Au@Fc-PBA) as the nanolabels for signal transduction, exhibiting specific affinity for V.P. In the presence of V.P., the aptamer on the Fe_3_O_4_@NMOF and PBA on the AuNP can both recognize V.P. specifically to form the capture probe–V.P.–nano label complex. The complex was immobilized on the surface of SPE for electrical signal measurements using a magnet. Under the optimal conditions (20 min incubation, 20 °C temperature, 7.5 pH, 2:1 for [Fc]/[PBA]), the sensor was demonstrated to have a high sensitivity (10–10^9^ cfu/mL for detection range, 3 cfu/mL for LOD) without a purification procedure. To improve the detection efficiency, Zhu et al. [78] proposed a dual-ratiometric electrochemical aptasensor to detect malathion (MAL) and omethoate (OMT) simultaneously, as shown in Figure 6E. The glassy carbon electrode (GCE, 3 mm) substrate was first modified with carbon nanohorns/anthraquinone-2-carboxylic acid/Au nanoparticles (CNHs/AQ/AuNPs). Then, hairpin DNA (hDNA) was immobilized on the CNHs/AQ/AuNPs via an Au-S bond. There were two independent and specific binding sites for corresponding aptamers of targets (MB-Apt1 for MAL and Fc-Apt2 for OMT). With the addition of MAL or OMT, the corresponding aptamers were released from the hairpin DNA, causing a change in the electrical current. This dual electrochemical aptasensor demonstrated a linear detection range of 3 pg/mL to 3 ng/mL with an LOD of 1.3 pg/mL for MAL and 10 pg/mL to 10 ng/mL with an LOD of 2.8 pg/mL for OMT.

#### 2.2.2. Competitive Assays for Biomolecules Based on Aptamer-Modified MP and AuNPs

**Figure 7:**
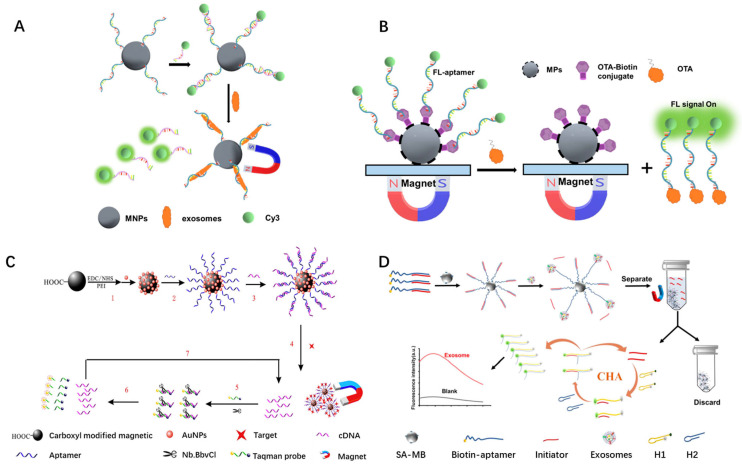
Competitive assay methods via fluorometric apta-biosensing (**A**) The competitive detection of exosomes based on an aptamer specific to the CD63 protein. (**B**) An aptasensor based on an aptamer-modified fluorescent particle for OTA detection. (**C**) Fluorescence signal-amplified strategy and procedure using AuNPs/MPs and a nicking enzyme, reproduced with permission from [82]. Copyright 2017 Analytica Chimica Acta. Numbers 1–7 represent the 7 steps used in the procedure. (**D**) AICHA signal amplification strategy for exosome detection, reprinted with permission from [83]. Copyright 2022 American Chemistry Society.

A competitive fluorescence detection method based on the release of aptamer-conjugated nanoparticles (NPs) or fluorophores in the presence of target biomolecules has also been utilized in biosensing. In the competitive assays, NPs or fluorophores are first conjugated with the aptamers. Target molecules then bind with aptamers with higher affinity; subsequently, the NPs or fluorophores are released due to their lower affinity. The released NPs or fluorophores are collected and detected in the supernatant. Yu et al. [79] established a simple fluorescent method for the quantitative detection of CD63 protein in the exosomes, as shown in Figure 7A. Aptamer-functionalized MNPs were hybridized with Cy3-labeled complementary sequence (CS)-aptamers. In the presence of exosomes, the CD63 protein bound with the aptamer with higher affinity, causing the release of Cy3-labeled complementary sequences into the supernatant. The concentration of exosomes can be back-calculated from the fluorescent intensity in the supernatant. This simple method achieved an LOD of 1.0 × 10^5^ particles/μL for exosome detection (detection range of 1.0 × 10^5^ to 1.0 × 10^9^ particles/μL). One problem of using the Cy3 fluorophore is the relatively low photostability [80] due to photobleaching. Hayat et al. [81] utilized carboxy fluorescent particles instead of fluorophores as the signal label, as shown in Figure 7B. Due to the larger surface area to volume, fluorescent particles generated a brighter/enhanced fluorescence signal than any fluorophore. Moreover, carboxy functional groups on fluorescent particles increased the immobilization efficiency of aptamers. In this design, biotinylated ochratoxin A (OTA)-modified MNPs were immobilized on the substrate, and aptamer-modified fluorescent particles were bound with OTA-biotin-modified MNPs. In the presence of free OTA, OTAs displaced the conjugated sites and bound with aptamer-modified fluorescent NPs, causing the detachment of aptamer-modified fluorescent NP–OTA complexes. The higher concentration of free OTA, the more detachment of fluorescent complexes. This sensor achieved a detection range of 0.2–140 nM with an LOD of 0.21 nM.

In addition to fluorescent particle-assisted signal enhancement, nicking enzyme-assisted signal amplification (NEASA) provides a simple strategy without any specialized instrumentation. Luo et al. [82] developed a sensitive aptasensor for ampicillin detection using a nicking enzyme, as shown in Figure 7C. MPs were coated by AuNPs with the help of polyethyleneimine. The AuNP/MP composites not only exhibited distinguished magnetic separation capacity but were also conjugated with aptamers via a strong covalent bond. Next, thiolated aptamers were bound to the surface of AuNPs via the Au-S bond, serving as anchors. CS-aptamers were added and hybridized with aptamers effectively. With the addition of targets (ampicillin), the binding between ampicillin and aptamers induced the release of the CS-aptamers. After separating functionalized MPs, CS-aptamers were collected and bound with the TaqMan probes labeled by 5-hexachlorofluorescein to form the duplex structure. The nicking enzyme cleaved the TaqMan probes into two pieces, resulting in fluorescence enhancement. Further, the released CS-aptamers hybridized with the remaining TaqMan probes to initiate the cycle of NEASA. The completion of the cycles generated a strong amplified fluorescence intensity. This enzyme-assisted aptasensor achieved the detection range of 0.1–100 ng/mL with an LOD of 0.07 ng/mL. Recently, catalytic hairpin assembly (CHA) was used as an enzyme-free technique for signal amplification with high sensitivity and selectivity. Zhou et al. [83] proposed the detection of exosomes using aptamer-initiated CHA (AICHA) fluorescence signal amplification, as shown in Figure 7D. In this strategy, target-specific aptamers hybridized with an initiator and conjugated to the surface of MNPs. In the presence of exosomes, the aptamers could recognize and bind to the exosomes due to the higher affinity between the aptamers and membrane proteins of exosomes, resulting in the release of initiators into the supernatant. Two hairpins (H1 labeled with a fluorophore and quencher and H2 with no labels) were involved in subsequent procedures. After isolating functionalized MNPs, the free initiators hybridized with H1, while the unpaired sequence of H1 complemented with H2. Due to the more thermodynamically favorable interaction between H1 and H2, the paired section between H1 and the initiator was replaced by the unpaired sequence of H2 to form the H1–H2 duplex, thus releasing the initiators that triggered the next circle of CHA and fluorescence recovery of fluorophores. These cycles ultimately generate numerous H1–H2 complexes, thereby leading to fluorescence signal amplification. This strategy was demonstrated for detecting MCF-6 cell-derived exosomes with a wide detection range from 8.4 particles/μL to 8.4 × 10^5^ particles/μL and an LOD of 0.5 particles/μL.

**Figure 8:**
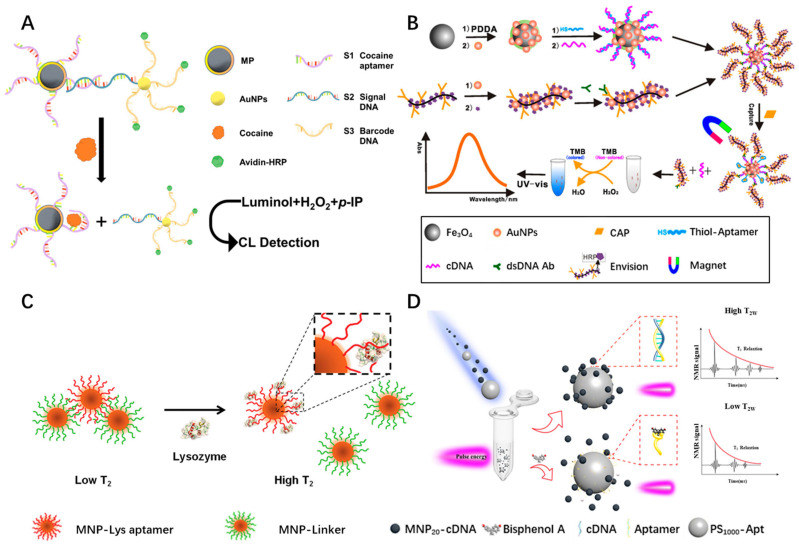
Competitive assay methods using chemiluminescence and magnetic relaxation switching (MRSw). (**A**) The chemiluminescence detection of cocaine using HRP-labeled aptamer and functionalized MPs. (**B**) The colorimetric aptasensor to detect CAP using ds-DNA Ab/EV-AuNPs-HRP as the signal tag, reproduced with permission from [85]. Copyright 2015 Sensors and Actuators B: Chemical. (**C**) The magnetic nanosensor for Lys detection based on MRSw, reprinted with permission from [87]. Copyright 2011 American Chemical Society. (**D**) PS-MRS sensor for the detection of BPA, reprinted with permission from [88]. Copyright 2021 American Chemical Society.

Colorimetric sensing based on chemiluminescence, with the advantages of a direct readout with naked eyes, was also developed for biosensing based on AuNP release. This strategy utilizes a catalytically chromogenic reaction of enzymes, which can lead to more sensitive detection. Li et al. [84] developed the sensitive detection of cocaine by using chemiluminescence, as shown in Figure 8A. Cocaine aptamers (S1) were immobilized on the surface of AuNP-coated MMP (MMP-AuNPs). AuNPs were functionalized by a signal aptamer (S2) that hybridized with cocaine aptamers (S1) and multiple barcode aptamers (S3) modified with horseradish peroxidase (HRP). In the presence of cocaine, cocaine preferred to bind with cocaine aptamers, causing the dissociation of gold probes (AuNP-barcode aptamer-HRP). HRP reacted with the substrate solution and induced a color change, which was quantitively detected using a BPCL ultraweak luminescence analyzer. The aptasensor achieved a linear detection range of 1 × 10^−9^ to 1 × 10^−8^ M and an LOD of 0.48 nM. To achieve higher sensitivity of the aptasensor, one needs to increase the ratio of the enzyme molecule (HRP) and target analyte so that a single target molecule can trigger the release of more enzyme molecules into the substrate solution. Miao et al. [85] proposed a high-sensitivity colorimetric aptasensor for chloramphenicol (CAP) detection, as shown in Figure 8B. In this design, aptamers and CS-aptamers were immobilized on the surface of AuNP-coated magnetic microparticles (AuMNPs). Next, multiple ds-DNA antibodies (which can bind with ds-DNA specifically) and horseradish peroxidase (HRP)-labeled AuNPs were immobilized on the Envision reagent (EV) as an enzyme-linked polymer nano-tracer. Numerous EVs were immobilized on the surface of modified AuMNPs via specific binding between ds-DNA and ds-DNA antibodies. HRP, which can catalyze various substrates, was used as a color tracer. In the presence of CAP, CAP is preferentially bound with aptamers, resulting in the release of CS-aptamers. Subsequently, EVs containing numerous HRP-catalyzed substrate solutions for color transition achieved signal amplification. The color transition was quantified via ultraviolet-visible spectroscopy. This aptasensor demonstrated a linear detection range of 0.05–100 ng/mL for CAP and an LOD of 0.015 ng/mL.

To simplify the procedures, a magnetic relaxation switching (MRSw) sensor was developed to detect target molecules in one step without multiple washing steps. This method also mitigated optical background interference from particles or biological matter [52,70,86]. Bamrungsap et al. [87] proposed an MRSw-based sensor for lysozyme (Lys) protein detection, as shown in Figure 8C. Aptamer-modified MNPs conjugated with CS-aptamer-functionalized MNPs. The aggregation of MNP–MNP complexes induced a strong local magnetic field, resulting in the dephasing acceleration of adjacent water protons and subsequently a smaller spin-spin relaxation time (T_2_). In the presence of targets (Lys), aptamers bound with targets preferentially, leading to the dissociation of complexes and an increase in T_2_. This sensor achieved a nanomolar range (0–1000 nM) for Lys detection and an LOD of 30 nM. To amplify the magnetic relaxation signal and improve the sensitivity, polystyrene microspheres (PS) with high monodispersity and good suspension stability were used in the MRSw sensor. These properties eliminated the effects of PS size and PS-induced non-target aggregation on T_2_ of the adjacent water protons. Huang et al. [88] designed an MRSw sensor based on polystyrene microspheres and MNPs (PS-MRS) for relaxation signal amplification, as shown in Figure 8D. Aptamer-functionalized PS conjugated with complementary aptamer (CS)-modified MNPs (MNP-cDNA) to form the complexes. The large area of PS facilitated the capture of magnetic particles, resulting in signal amplification. With the addition of bisphenol A (BPA), aptamers could bind with BPA with higher affinity to form PS–aptamer–BPA complexes and free MNP-cDNA. Under the optimized conditions, this MRSw sensor achieved a detection range of 0.1–100 ng/mL and an LOD of 0.06 ng/mL.

### 2.3. Biomolecule Detection with Resistive Pulse Sensor via Aptamer-Modified Nanoparticles

**Figure 9:**
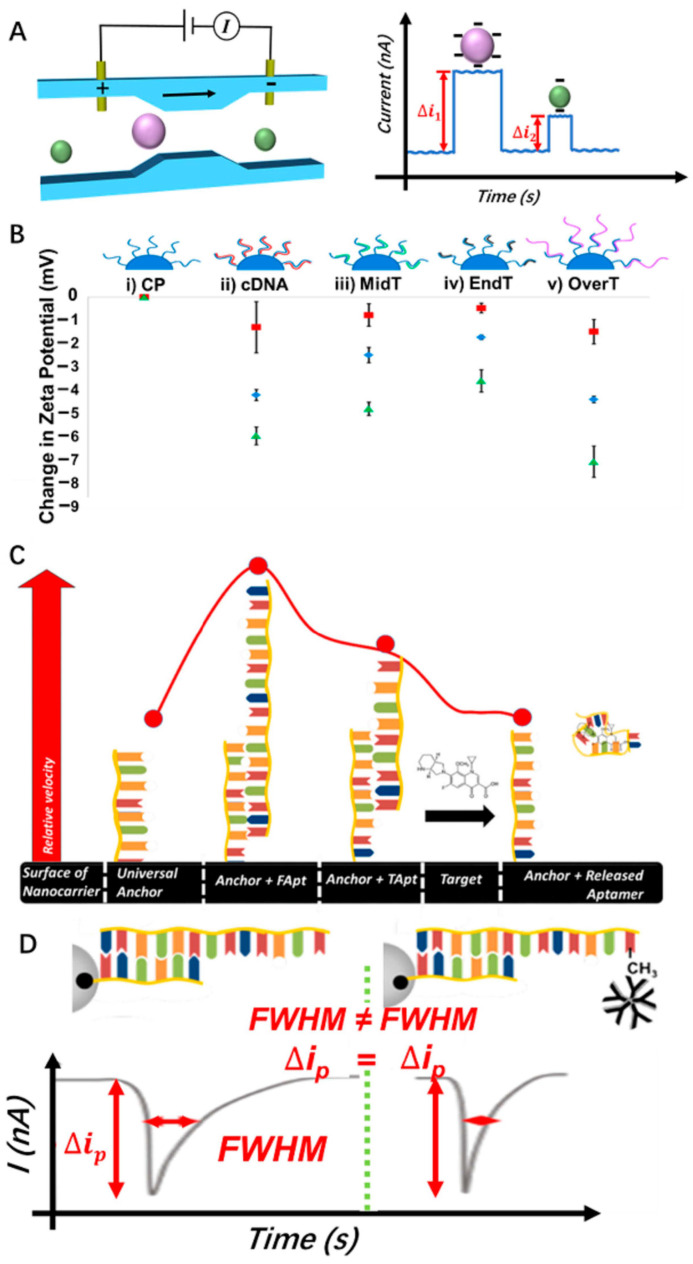
RPS—aptasensors based on an aptamer capture assay. (**A**) Schematic of the resistive pulse setup. (**B**) Schematic of different kinds of CS-conjugated aptamers via different binding positions and lengths, reproduced with permission from [94]. Copyright 2016 American Chemical Society. (**C**) Schematic of a displacement assay for small molecule detection, reproduced with permission from [95]. Copyright 2021 Talanta. (**D**) Assessment of site-specific DNA methylation by RPS, reproduced with permission from [96]. Copyright 2018 American Chemical Society.

The above-mentioned methods require measuring the fluorescence/light intensity change or magnetic-time relation change in a bulk solution. In the past decade, resistive pulse sensors (RPSs) have demonstrated significant advantages for detecting micro- and nanoscale bio-objects. RPS, or a Coulter counter, typically consists of a micro/nanopore/channel connecting two fluidic reservoirs. An electric field is applied across a pair of electrodes placed on both sides of the micro/nanopore. The passage of a micro/nano object distorts the electric field and causes a resistance change of the pore/channel, as shown in Figure 9A. With a simple structure, RPS can analyze individual target analytes yet provide comprehensive information (e.g., size, surface charge, mobility) with high resolution. In principle, RPS can analyze targets with single-molecule resolution. While RPSs can only identify analytes via their physical properties (e.g., size, surface charge, mobility, shape, etc.), their detection specificities are low. Despite this, RPSs have been used in combination with the antibody recognition method for detecting proteins [89,90]. One problem with antibody-based recognition is the random orientation of antibodies on a solid surface and desorption by the change in ionic strength or pH, which may affect specificity, sensitivity, and stability in biosensing [91,92,93].

#### 2.3.1. RPS—Aptasensors Based on an Aptamer Capture Assay

Aptamers have high specificity and sensitivity against target analytes [93,97]. They can be chemically synthesized and modified with high stability in a harsh working environment. They can also undergo conformational changes in response to target analytes or their complementary sequences [98,99,100]. Hence, by incorporating aptamers into RPS, the sensor’s capabilities have been expanded to detect not only proteins but also small biomolecules, metal ions, DNA sequencing, etc. [101,102,103]. In this strategy, aptamer-modified nanocarriers (magnetic or polystyrene NPs) can assist in the separation of functionalized target NPs from the bulk solution or serve as capture probes. By combining RPS and these aptamer-modified nanocarriers, the fast, specific, and selective detection of various nanoobjects has been demonstrated with high sensitivity and robustness.

Blundell et al. [94] used the tunable resistive pulse sensing (TRPS) technique to demonstrate the particle-by-particle charge analysis of polystyrene NPs (200 nm), as shown in Figure 9B. Aptamer (with 25 bases in length)-functionalized nanoparticles served as capture probes. The complementary sequences (CSs) were designed to bind to capture probes with different lengths and positions of the complementary sections (e.g., cDNA for full complementary binding, MidT for binding to the middle of the capture probe, EndT for binding to the end of capture probe, OverT for binding to the end of capture probe and overhanging a section of CS in the solution) via molecular hybridization. After hybridization, the nanoparticle–DNA–CS complexes served as the signal transducers that could be detected and analyzed by the TRPS. The partial or full hybridization between the capture probe and CS (different lengths) contributed to a variation in zeta potential before and after hybridization. The variations in the zeta potential of nanoparticles caused a mobility or translocation time change of NPs, which was detected by the TRPS when the NPs passed through the sensing pore. Further, due to the specific recognition between aptamers and target analytes, the presence of target analytes can trigger the conformational change of aptamers, which would also induce a change in the mobility or translocation time. Thus, this method can be used to identify the target biomolecules in terms of RPS measurements. Maugi et al. [95] modified MNPs (mode diameter 120–150 nm) with single-stranded DNA (ssDNA), which served as anchors, as shown in Figure 9C. Aptamers were added to form a stable double-stranded DNA (dsDNA) complex due to the complementary design. After the targets were added, a conformational change occurred where aptamers preferred to bind with targets due to higher affinity, which induced the release of aptamers from the NPs. After the release, only ssDNA was left on the NP surface, resulting in a change in surface charge and a subsequent change in mobility/translocation time. RPS was used to measure the change in the translocation time of NPs before and after the aptamer release, from which the target concentration can be determined. Three targets (Moxifloxacin, Imatinib, and Irinotecan) were used to demonstrate this strategy. The results showed a measurable difference in the translocation time change, resulting from surface charge variation. Healey et al. [96] presented an aptamer assay integrated with TRPS to quantify the interactions between DNA and proteins (e.g., determine the specific sites of DNA methylation (antibody binding sites)), as shown in Figure 9D. Aptamers (a shorter DNA sequence) and CS (a longer DNA sequence) were hybridized at a complementary region; 120 nm magnetic NPs were modified by dsDNA and served as capture probes. Due to the longer CS, the non-hybridizing sequences with methylation sites were hanging in the solution; target antibodies tended to bind with the methylation sites. The TRPS was used to monitor the velocity/mobility of nanoparticles through the sensing pore that indicated the number and location of antibodies bound with probe DNAs. CSs with different positions and numbers of methylation sites were used to validate the sensor (i.e., single methylated site (MidDNA), single methylation (EndDNA), and two methylation sites (DoubleDNA)). As a result, an obvious decrease in velocity after antibody binding was observed. Furthermore, the specific sites and the number of binding sites of antibodies also affected the zeta potential and hence the mobility of NPs due to two factors: (1) the binding of DNA and proteins changed the DNA structure, causing a conformational change of DNA into a tertiary structure, which requires an increasing number of counter ions to stabilize; (2) the binding disrupted the double-layer structure of the DNA backbone and affected the electrophoretic mobility.

#### 2.3.2. RPS—Aptasensors Based on Aptamer Folding

**Figure 10:**
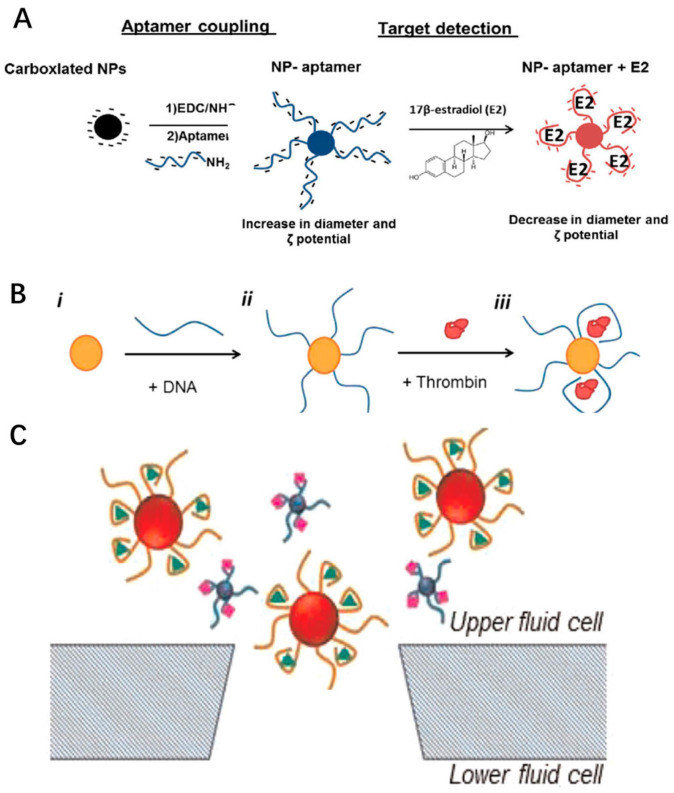
RPS—aptasensors based on aptamer folding for biomolecule detection. (**A**) Aptamer-based sensor for 17β-estradiol detection via the size contraction response of aptamer-functionalized nanoparticles, reproduced with permission from [104]. Copyright 2014 Biosensors and Bioelectronics. (**B**) AptaTRPS based on aptamer-tagged nanoparticles to monitor the interaction between the aptamer and protein, reproduced with permission from [105]. *i*, *ii*, *iii* represent bare NPs, NPs modified by aptamers, and modified NPs interacted with targets respectively. Copyright 2014 American Chemistry Society. (**C**) Size-multiplexed detection of biomolecules using aptamers and TRPS, reproduced with permission from [106]. Copyright 2015 Biosensors and Bioelectronics.

Alsager et al. [104] demonstrated a simple sensor by utilizing aptamer-functionalized 217 nm NPs and TRPS for 17β-estradiol detection, as shown in Figure 10A. Aptamer-modified nanoparticles served as signal transducers. After modification, the diameter and zeta potential of NPs increased. The presence of the target (17β-estradiol) induced a more tightly folded aptamer conformation on the surface of NPs. A distinct decrease in the diameter of the aptamer-functionalized NPs and less negative zeta potential were observed by TRPS, which were correlated with the target concentration. This sensor was able to detect 17β-estradiol at the nanomolar level in the buffer solution. To demonstrate the excellent selectivity of the TRPS sensor based on aptamer recognition, Billinge et al. [105] utilized TRPS to monitor interactions between several different aptamers (Thrombin-15, Thrombin-Marray, and ThrombinEvol) and the target (thrombin protein), as shown in Figure 10B. Aptamer-modified 128 nm MNPs were used as capture probes. In the presence of thrombin protein, only aptamers containing a G-quadruplex structure (Thrombin-15 and Thrombin-Marray) underwent the conformational change, resulting in the shielding/folding of the polyanion backbone of aptamers. As a result, translocation time changes of functionalized magnetic NPs were observed by the TPRS. The concentration of the thrombin could thus be determined. This work demonstrated the tag-less detection of thrombin at a nanomolar level (0–200 nM). To improve the detection efficiency of the sensor and decrease the assay time, Billinge and Platt [106] utilized two different aptamers (PDGF-BB aptamer and VEGF aptamer) to modify two types of superparamagnetic NPs (120 nm for VEGF aptamer and 300 nm for PDGF-BB aptamer) for the multiplexed detection of targets (VEGF and PDGF), as shown in Figure 10C. Upon adding the target molecules, VEGF and PDGF were captured by their respective targets due to specific binding, causing aptamers to fold due to a conformational change that shields the surface charge. The variations in surface charge resulted in varied mobility of NPs. In one-step analysis, one can monitor the changes in the translocation time of two functionalized magnetic nanoparticles simultaneously. The measurements of two different targets can be easily separated based on the sizes of the carrier NPs. The size-multiplexed sensor demonstrated the detection of targets at the nanomolar level (0–200 nM).

#### 2.3.3. RPS—Aptasensors Based on NP Release

**Figure 11:**
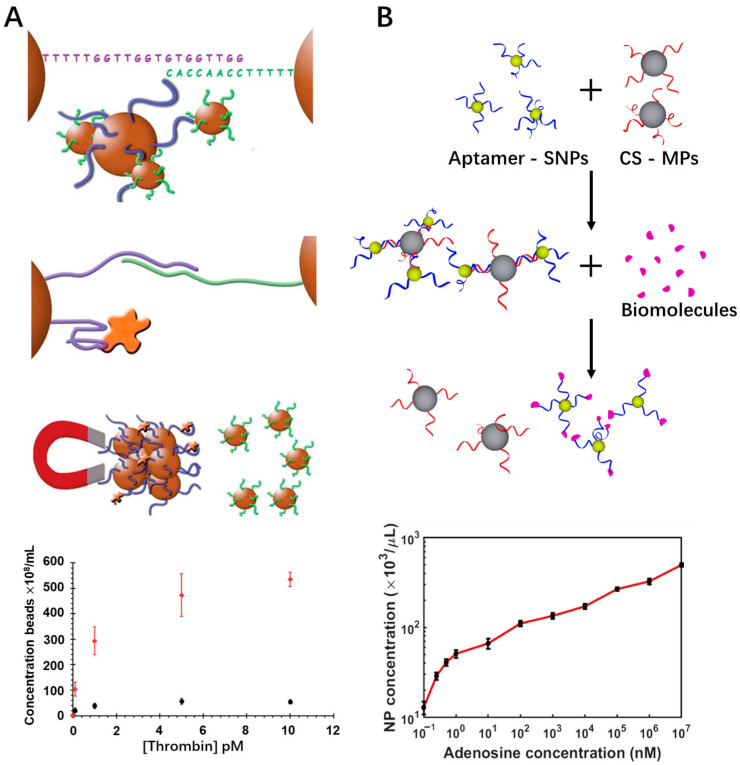
RPS—aptasensors based on NP release. (**A**) Aptamer-based dispersion assay using TRPS for thrombin detection, reproduced with permission from [107] Copyright 2015 Royal Society of Chemistry. (**B**) Mechanism of adenosine detection via aptamer reconfiguration and nanoparticle counting, reproduced with permission from [108]. Copyright 2022 Biosensors and Bioelectronics.

To improve the sensitivity of the aptasensor to the picomolar level, Billinge and Platt developed a highly sensitive platform to detect thrombin based on NP release, i.e., disrupting MP–NP aggregates using target molecules, as shown in Figure 11A [107]. Aptamer-modified 1 μm magnetic micro-beads and complementary sequence (CS) aptamer-modified 400 or 800 nm carboxyl NPs were used to demonstrate the principle. These two-sized NPs tended to bind with 1 μm magnetic beads and form 1 μm–400 nm and 1 μm–800 nm complexes via aptamer recognition. The complexes were then separated from the solution using an external magnet. In the presence of thrombin, the aptamer underwent a conformational change, causing the release of NPs. After isolating the magnetic beads, the remaining NPs were counted by TRPS. As the thrombin and the remaining carboxyl NPs maintain a positive correlation, the thrombin concentration can be obtained from the counts of the remaining NPs. Note that the sensitivity of the assay is ascribed to the disruption of multiple complexes by the target protein. This method exhibited a high sensitivity. However, due to the limited binding capacity (1 μm–400 nm and 1 μm–800 nm), the detection range was narrow (up to 10 pM). The detection range can be further improved by attaching a large number of smaller NPs to a larger microparticle surface. Xu et al. [108] proposed an ultrasensitive sensor for adenosine detection using aptamer-based molecular recognition and RPS, as shown in Figure 11B. Aptamer-modified 500 nm NPs were attached to CS-conjugated 5 μm magnetic carriers to form the complexes. With the addition of target molecules (adenosine), nanoparticles were released from the microcarriers due to the conformational change of aptamers. The released nanoparticles were collected and detected by a solid-state sensing channel of 2 μm × 2 μm × 10 μm (width × height × length). Instead of TPRS, the PDMS-based solid sensing channel can quantify the size and concentrations of nanoparticles more accurately, with less deformation without mechanical stretching. Due to the decent binding capacity (large surface-to-volume ratio), approximately 44 NPs were attached to one microparticle. The large number of attached NPs assisted in adenosine detection with a wider detection range of 0.1 nM to 10 mM with an LOD of 0.168 nM (44.85 pg/mL). The method exhibits the potential for small biomolecule detection at an ultra-low abundance.

The performance matrix of all nanoparticle-based aptasensors, as well as their major advantages and limitations, is summarized in Table 1. It is worth mentioning here that LSPR and SERS have been used for the detection and characterization of biomolecules. Table 2 provides the performance matrix of LSPR and SERS for detecting various biomolecules. For a comparison, the performance matrix of the nanoparticle-based aptasensors for detecting the same types of molecules is also given in Table 2. From Table 2, nanoparticle-based aptasensors typically have a wider detection range and a lower LOD.

Note that nanomaterials play a critical role and are increasingly being used in biosensing applications due to their unique physicochemical properties, such as a high surface-to-volume ratio, high reactivity, and size-dependent optical and electronic properties. There are several excellent review articles [29,123,124,125] that summarized the recent nanomaterial development for various biosensing applications. Recent advances and future directions of these nanomaterial developments can be found in these references.

## 3. Conclusions and Future Outlook

Aptamers with high stability, binding affinity, and selectivity for their targets have become powerful probes for biosensing. Nanoparticles, serving as carriers for signal transducers or capture probes, facilitate the signal amplification, immobilization, and separation of target biomolecules. Biosensors integrating aptamers and nanoparticles are used widely for detecting a variety of bio-objects, such as nucleic acids, proteins, lipids, and metabolites, with high sensitivity and affinity.

Despite the promises, one challenge for NP-based aptasensors is the suitability for on-site testing in diverse environments. Most of the aforementioned works were carried out in a controlled lab environment. As the binding affinity and specificity against target analytes are influenced by environmental factors, such as pH, temperature, ionic strength, etc., translating these assays into broad applications (e.g., point-of-care testing) relies on developing more stable aptamers that are unsusceptible to the environment-influenced structural changes while still having high affinity for targets.

The second challenge is to detect multiple biomolecules using aptasensors. Multiple specific aptamers must be used to functionalize the surfaces of nanomaterials. Designing specific sequences of aptamers that enable specificity to only one type of target biomolecules and eliminate nonspecific interactions is critical for developing aptasensors.

It is worth mentioning that RPS-based aptasensors exhibit ultra-sensitive detection for biomolecules. In principle, these sensors can achieve unprecedented single-molecule resolution. However, one long-standing limitation of RPS is its low throughput. To detect nanoscale biomolecules, the sensing channel must be scaled down to the target size to obtain a decent signal-to-noise ratio. As a result, only a small volume of the target sample can be analyzed in a given time. Thus, multiplexed detection becomes necessary. Han et al. [126] and Billinge & Platt et al. [106] used different-sized micro/nanoparticles modified by probe molecules to detect two different biomolecules. As the magnitude of an RPS signal is proportional to the volume of the carrier MP/NP occupied in the sensing pore, signals of the different target molecules can be separated in terms of signal magnitudes (particle sizes). However, this method can only work to multiplex signals from limited types of carrier particles with different sizes. Several researchers have developed devices with multiple sensing channels to demonstrate high-throughput detection. Song et al. [127] proposed a space modulation sensor with eight peripheral sensing channels connected to a central reservoir for microparticle detection. This sensing principle can be extended to nanoparticle counting. However, while the throughput was multiplied by many folds, each sensing channel can be considered an individual RPS due to independent detection electronics. Adding a large number of sensing channels along the periphery is impractical. Jagtiani et al. [128] demonstrated frequency modulation on a parallel resistive pulse sensing array. While only one combined signal needs to be measured, the sensor needs to operate in a resistance-dominant range. This limits the number of sensing channels that can be used within this narrow frequency range. Liu et al. [129] proposed a code modulation sensor for the counting of microparticles. However, complex coplanar electrode patterns need to be fabricated inside the RPS channels, which is necessary for encoding signals from the parallel RPS channels. However, it is challenging to fabricate sets of electrodes with complex patterns within a nanopore or nanochannel for sensitive biomolecule detection. This multiplexing method also has difficulty in accurately decoding the combined signals based on the correlation coefficient [130,131,132]. Xu et al. [133] reported a geometry modulation RPS sensor with simple measurement electronics for microparticle counting. The electrical signal from each RPS channel was encoded by a specific waveform generated by the unique geometry of each sensing channel. Only a DC power source and a pair of electrodes were needed for the measurement. This design achieved high accuracy in counting and sizing microparticles. This RPS design has the potential for high-throughput nanoparticle counting and sizing, which can be combined with aptamer recognition for ultrasensitive and ultra-large-range biomolecular detection. This RPS can be further improved by designing simplified but unique electrode patterns or channel geometries while still being able to encode the RPS signal from each sensing channel.

## Figures and Tables

**Table 1:** Performance matrix of all the reviewed work.

Target Analyte	Sensing Type	Detection Range	LOD	Merits	Limitations	Ref.
Histamine	Col	up to 2000 nM	8 nM	Simple, low cost	Incomplete dissociation of nontarget binding fragments of aptamers	[37]
Vitamin D3	Col	1–1000 nM	1 nM	Sufficient dissocitation of residual binding	Nontarget aggregation	[41]
E2	Col	100–10,000 nM	500 nM	Significant suppression of nontarget aggregation	Cannot be reused	[42]
Dopamine	Col	0.1–10 mM	2.1 mM	Reusable platform	Low sensitivity	[43]
Pesticide malathion	Col	5 pM–10 nM	1 pM	Lower detection range and high sensitivity	Long incubation time	[44]
Interleukin-6	Col	3.3–125 μg/mL	1.95 μg/mL	Rapid detection	Low sensitivity	[45]
AMP	Fl	0.04–20 μM	18 nM	High sensitivity	Need recovery of QDs	[49]
AFB1	Fl	61 pM–4.0 μM	61 pM	Lower limit of detection	Long detection time	[50]
rHUEPO-α	Fl	0–1600 nM	0.92 nM	High sensitivity	Long incubation time	[51]
Caspase-3	Fl	0.01–10 ng/mL	10 pg/mL	High sensitivity	The sensitivity will be affected by the distance between the position of the fluorophore and detector	[55]
PfLDH	Fl	0.3 ng/mL–300 μg/mL	0.3 ng/mL	Fixed sensing platform, high sensitivity, rapid detection	Bulky optical instrumentation	[56]
PV	Col and Fl	2.5–20 μg/mL for Col 2.38–40 μg/mL for Fl	0.72 μg/mL	High sensitivity, easy and fast operation	Narrow detection range and photobleaching	[57]
PCT	Fl	7.6 pg/mL–125 ng/mL	3.8 pg/mL	High stability and sensitivity	Bulky optical instrumentation	[59]
α-thrombin HNE	Fl	0.002–10 pM 1–1000 pM	2 fM 100 fM	High sensitivity	Long enzyme reaction time	[63]
Thrombin	Fl	up to 4 μg/mL	10 ng/mL	Short incubation and reaction time	Low sensitivity	[64]
pLDH	Fl	10 aM–1.5 fM	10 aM	Ulrasensitivity	Bulky optical instrumentation	[65]
S. typhi	Fl	10–10^7^ cfu/mL	13.6 cfu/mL	High specifity and sensitivity	Bulky optical instrumentation	[66]
OTC and KAN	Col	10^−6^–10^5^ pg/mL	1 ag/mL	Wide detection range and low LOD	Background interference	[67]
V. parahaemolyticus	Col	10–10^6^ cfu/mL	10 cfu/mL	High sensitivity	Background interference	[68]
MCF-7	Col	10–100,000 cells/mL	3 cells/mL	High sensitivity and low LOD	Background interference	[69]
α-thrombin	MRSw	1.6–30.4 nM	1.0 nM	Without the interference of background	Sophisticated instrumentations	[71]
Hg^2+^	MRSw	10 nM–5 μM	2.7 nM	Wide detection range	Sophisticated instrumentations	[72]
Vibrio alginolyticus	MRSw	4–4 × 10^3^ cfu/mL	26 cfu/mL	High sensitivity	Need to optimize some factors to affect the sensitivity	[73]
MO	EC	10^−17^–10^−12^ M	3.3 aM	Sensitive, portable, cost-efficient, and fast analysis	Non-reusable	[75]
BNP	EC	1–10,000 pg/mL	0.56 pg/mL	Wide detection range	Non-reusable	[76]
V.P	EC	10–10^9^ cfu/mL	3 cfu/mL	Rapid and on-site quantification	Non-reusable	[77]
MAL OMT	EC	3 pg/mL–3 ng/mL 10 pg/mL–10 ng/mL	1.3 pg/mL 2.8 pg/mL	Multiple detection	Non-resuable	[78]
CD63	Fl	1.0 × 10^5^–1.0 × 10^9^ particles/μL	1.0 × 10^5^ particles/μL	Wide detection range	High LOD, low photostability	[79]
Ochratoxin A	Fl	0.2–140 nM	0.21 nM	High sensitivity	Bulky optical instrumentation	[81]
Ampicillin	Fl	0.1–100 ng/mL	0.07 ng/mL	High sensitivity	Enzyme-assisted technique	[82]
MCF-6	Fl	8.4–8.4 × 10^5^ particles/μL	0.5 particles/μL	Wide detection range	Bulky optical instrumentation	[83]
Cocaine	Col	1.0 × 10^−9^ –1.0 × 10^−8^ M	0.48 nM	Simple instrumentation	Narrow detection range	[84]
CAP	Col	0.05–100 ng/mL	0.015 ng/mL	High sensitivity	Sophisticated detection procedures	[85]
Lys	MRSw	up to 1000 nM	30 nM	One-step detection	Low sensitivity	[87]
BPA	MRSw	0.1–100 ng/mL	0.06 ng/mL	High sensitivity	Sophisticated instrumentations	[88]
Moxifloxacin Imatinib Irinotecan	RPS	up to 200 μΜ up to 13 μΜ up to 7 μΜ	–	Wide detection range	Low resolution	[95]
17β-estradiol	RPS	up to 400 nM	–	Label-free detection	Narrow detection range	[104]
Thrombin	RPS	up to 200 nM	–	Label-free detection	Narrow detection range	[105]
VEGF PDGF	RPS	up to 2 nM up to 10 nM	–	Multiplexed detection for different biomolecules	Narrow detection range	[106]
Thrombin	RPS	up to 10 pM	–	High sensitivity	Narrow detection range	[107]
Adenosine	RPS	0.1 nM–10 mM	0.168 nM	Ultrahigh sensitivity and wide detection range	Low throughput	[108]

Note: in the Table, EC, electrochemical; Col, colorimetric; Fl, fluorescent; MRSw, magnetic relaxation switching; RPS, resistive pulse sensing.

**Table 2:** Summary of biomolecular detections based on LSPR and SERS.

Target Analyte	Method	Detection Range	LOD	Ref.
Cardiac troponin I	LSPR	up to 20 ng/mL	ca. 30 pM	[109]
Alanine aminotransferase	LSPR	10–1000 U/L	10.61 U/L	[110]
Acetylcholine	LSPR	up to 1000 μM	14.28 μM	[111]
Cholesterol	LSPR	up to 10 mM	1.131 mM	[112]
Aflatoxin B1	LSPR	up to 20 ng/mL	0.18 ng/mL	[113]
p-cresol	LSPR	up to 1000 μM	57.43 μM	[114]
Histamine	LSPR	0.23–10.23 mg/kg	1.0 mg/kg (0.0089 mM)	[115]
Creatinine	SERS	0.0000056–0.001 M	5 × 10^−6^ M	[116]
Cardiac troponin I	SERS	0.01–1000 ng/mL	5 pg/mL	[117]
Cocaine	SERS	1–5000 ng/mL	1 ng/mL	[118]
H_2_O_2_ Cholesterol	SERS	up to 100 μM	5.5 × 10^−6^ M 3.7 × 10^−6^ M	[119]
Ochratoxin A Aflatoxin B1	SERS	10^3^–10^−5^ μg/mL	2.63 pg/mL 4.15 pg/mL	[120]
Glucose	SERS	0.5–10 mM	0.1 mM	[121]
Histamine	SERS	10^−8^–10^−3^ mol/L	10^−8^ mol/L	[122]
Histamine	Col	up to 2000 nM	8 nM	[37]
Dopamine	Col	1–1000 nM	1 nM	[43]
AFB1	Fl	0.04–20 μM	18 nM	[49]
OTA	Fl	0.2–140 nM	0.21 nM	[81]
Ampicillin	Fl	0.1–100 ng/mL	0.07 ng/mL	[82]
Cocaine	Col	1.0 × 10^−9^–1.0 × 10^−8^ M	0.48 nM	[84]

Note: in the Table, EC, electrochemical; Col, colorimetric; Fl, fluorescent; MRSw, magnetic relaxation switching; RPS, resistive pulse sensing.

## Data Availability

Not applicable.
